# LPS-inducible circAtp9b is highly expressed in osteoporosis and promotes the apoptosis of osteoblasts by reducing the formation of mature miR-17-92a

**DOI:** 10.1186/s13018-022-03072-x

**Published:** 2022-03-28

**Authors:** Yinan Feng, Li Ding, Longguang Li

**Affiliations:** 1 Department of Endocrine and Metabolism, The Third Affiliated Hospital of Jinzhou Medical University, Jinzhou City, 121000 Liaoning Province People’s Republic of China; 2 Department of Respiratory, The Third Affiliated Hospital of Jinzhou Medical University, Jinzhou City, 121000 Liaoning Province People’s Republic of China; 3 Rehabilitation Department, The Third Affiliated Hospital of Jinzhou Medical University, No.2, Section 5, Heping Road, Linghe District, Jinzhou City, 121000 Liaoning Province People’s Republic of China

**Keywords:** Osteoporosis, circAtp9b, miR-17-92a, Maturation, Apoptosis

## Abstract

**Background:**

Circular RNA circAtp9b is an enhancer of LPS-induced inflammation, which promotes osteoporosis (OS). This study explored the role of circAtp9b in OS.

**Methods:**

RT-qPCR was performed to detect the expression of circAtp9b and microRNA (miR)-17-92a (both mature and premature) in OS and healthy controls. The subcellular location of circAtp9b was assessed by nuclear fractionation assay. The direct interaction between circAtp9b and premature miR-17-92a was detected by RNA pull-down assay. The role of circAtp9b in regulating the maturation of miR-17-92a in osteoblasts was explored by overexpression assay and RT-qPCR. Cell apoptosis was analyzed by cell apoptosis assay.

**Results:**

OS patients exhibited upregulation of circAtp9b and premature miR-17-92a, but downregulation of mature miR-17-92a. In osteoblasts, circAtp9b suppressed the maturation of miR-17-92a. LPS upregulated circAtp9b and premature miR-17-92a, and downregulated mature miR-17-92a in osteoblasts. CircAtp9b was detected in both nucleus and cytoplasm, and it directly interacted with premature miR-17-92a. Overexpression of circAtp9b reduced the effects of miR-17-92a on the apoptosis of osteoblasts induced by LPS.

**Conclusion:**

CircAtp9b is LPS-inducible and upregulation of circAtp9b in OS promotes the apoptosis of osteoblasts by reducing the formation of mature miR-17-92a.

**Supplementary Information:**

The online version contains supplementary material available at 10.1186/s13018-022-03072-x.

## Background

As a bone condition characterized by the brittle and weak bones, osteoporosis (OS) is caused by the decreased bone density [1]. OS is not only affected by aging, but also closely correlated with hormones, conditions of nutrition and physical activities [2, 3]. It is estimated that more than half of women older than 50 years old will experience OS-related fracture [4]. Currently, clinical therapeutic methods for OS mainly include hormone replacement, calcium supplement, and inhibition of bone resorption. However, due to the toxic and side effects of drugs, poor absorption and poor patient compliance, the treatment effect is not satisfactory. Therefore, novel preventative and treatment approaches are needed to improve the life quality of OS patients.

Previous studies on the molecular pathogenesis of OS have revealed the involvement of molecular and cellular mechanisms in bone gain or loss [5–7]. It has been reported that the Wnt/*β*-catenin signaling pathway plays a vital role in bone homeostasis by promoting osteoblast generation and inhibiting osteoclast differentiation [8]. Moreover, TNF-α, RANKL, or IL-1 activated NF-κB signal pathway can induce differentiation expression of genes in osteoclasts, prolong the life of osteoclasts and promote bone absorption [9]. However, targeted therapy for OS remains under research and more effective targets are needed [10, 11]. Circular RNAs (circRNAs) are self-ligated RNA transcripts that do not encode proteins but participate in human diseases mainly by affecting protein synthesis [12, 13]. Altered expression of circRNAs is frequently observed in OS patients, suggesting their involvement in OS and the potential role of these circRNAs as targets for OS treatment [14]. CircRNA circAtp9b is an enhancer of LPS-induced inflammation. It is known that silencing of circAtp9b alleviates inflammation induced by LPS [15], and LPS-induced inflammation contributes to OS [16]. Our preliminary sequencing analysis revealed altered expression of circAtp9b in OS and its inverse correlation with microRNA (miR)-17-92a. MiR-17-92a has been reported to be upregulated by estrogen and its overexpression targets Bim to suppress cell apoptosis in OS [17]. This study explored the role of circAtp9b in OS and its interaction with miR-17-92a.

## Materials and methods

### Patients and tissue samples

Plasma samples were donated by a total of 42 OS patients and 42 healthy controls at the Third Affiliated Hospital of Jinzhou Medical University. This study was approved by the Ethics committee of this hospital (approval number FB-2018-16). All procedures were following the guidelines of the Third Affiliated Hospital of Jinzhou Medical University and performed in accordance with the 1964 Declaration of Helsinki and its later amendments. The inclusion criteria were as follows: natural menopause after 40 years of age and a bone mineral density (BMD) of at least 2.5 standard deviation (SD) below the peak mean BMD of healthy young women (− 2.5 T-score) at the lumbar spine, total hip or femoral neck. Patients with a medical history of OP treatment, hormone replacement therapy, early menopause (< 40 years old), abnormal menopause, acute gastrointestinal inflammation, or chronic renal failure were excluded. All patients signed the informed consent. Clinical data of all patients and the controls are listed in Table 1.

**Table 1:** Summary of clinical data of 42 OS patients and 42 controls

	OS (I = 42)	Control (*n* = 42)
Gender female	32	32
Age (years, mean ± SD)	57.9 ± 8.9	58.2 ± 8.7
Obesity	23	13
Disease duration (months ± SD)	59.1 ± 14.1	NA
*Stage*		
III	16	NA
IV	26	NA
Smokers %	19	19
Drinkers %	26	25

### Osteoblasts and transfections

The cell model of OS in this study was osteoblasts isolated from an adult OS patient (406-05A, Sigma-Aldrich). Cells were cultivated in a 6-well cell culture plate containing DMEM medium supplemented with 2 mM l-glutamine and 1000 units of penicillin/streptomycin. Cells used in the subsequent assays were collected from passage 5 to 10. Incubation with medium containing 0, 2, 4, 6, 8 and 10 μg/ml LPS was performed for 48 h to achieve LPS treatment.

Overexpression of circAtp9b and miR-17-92a was achieved in osteoblasts by transfecting osteoblasts with either mimic of miR-17-92a or circAtp9b expression vector (pcDNA3.1, Invitrogen) through Neon™ Transfection System (Thermo Fisher Scientific)-mediated transfections. The same method was also used to transfect circAtp9b siRNA (Invitrogen). After transfection, cells were cultivated in fresh medium for another 48 h.

### Preparation of RNA samples

Total RNAs were isolated from both tissue and cell samples using Direct-zol (ZYMO Research). Following DNA digestion with DNase I (Invitrogen), RNA quality and concentration of all RNA samples were analyzed by BiOanalyzer. A RIN value higher than 8 was achieved in all samples.

### RT-qPCR analysis of gene expression

The preparation of cDNA samples was performed through reverse transcriptions (RTs) with about 2500 ng total RNAs as the template. The expression of circAtp9b and miR-17-92a (mature and premature) was determined by performing qPCRs with 18S rRNA and U6 as the internal control, respectively. Ct values were normalized using the 2^−ΔΔCT^ method.

### RNA pull-down assay

Premature miR-17-92a and NC RNA were labeled with biotin, and the labeled RNAs were named as Bio(pre)- miR-17-92a and Bio-NC, respectively. Labeled RNAs were transfected into osteoblasts through the methods mentioned above. Cells were harvested 48 h later, and cell lysis was performed. Magnetic beads were used to pull-down the complex. After that, RNA isolations were performed, followed by RT-qPCRs to determine to the expression of circAtp9b. Data normalization was performed through the same method mentioned above.

### Subcellular fractionation assay

Nuclear and cytoplasm samples were prepared using the Cytoplasmic and Nuclear RNA Purification Kit (Norgen, Ontario, Canada). Centrifugation (2400 g for 15 min) was performed to separate these two samples. After that, total RNAs were isolated from these two samples, followed by RT-PCRs to amplify circAtp9b. PCR products were subjected to 2.0% agarose gel electrophoresis and stained by EB. Images were taken by MyECL imager.

### Cell apoptosis assay

Osteoblasts were harvested after transfections, followed by incubation in DMEM medium containing 10 μg/ml LPS. Incubation was performed for 48 h, followed by 0.25% trypsin digestion. After that, cells were resuspended in binding buffer, and then, incubation with FITC and propidium iodide (PI) was carried out for 15 min in dark. Finally, flow cytometry was performed to analyze cell apoptosis.

### Statistical analysis

Two and multiple independent groups were compared by unpaired t test and ANOVA Tukey’s test, respectively. Normality of data distribution and homogeneity of variance were tested for all data. Normally distributed data were expressed as the mean ± standard deviation (SD). Data without normal distribution or homogeneity of variance were expressed as interquartile range. Correlations were subjected to Pearson’s correlation coefficient analysis. *p* < 0.05 was statistically significant.

## Results

### The expression of circAtp9b and miR-17-92a in OS

**Fig. 1:**
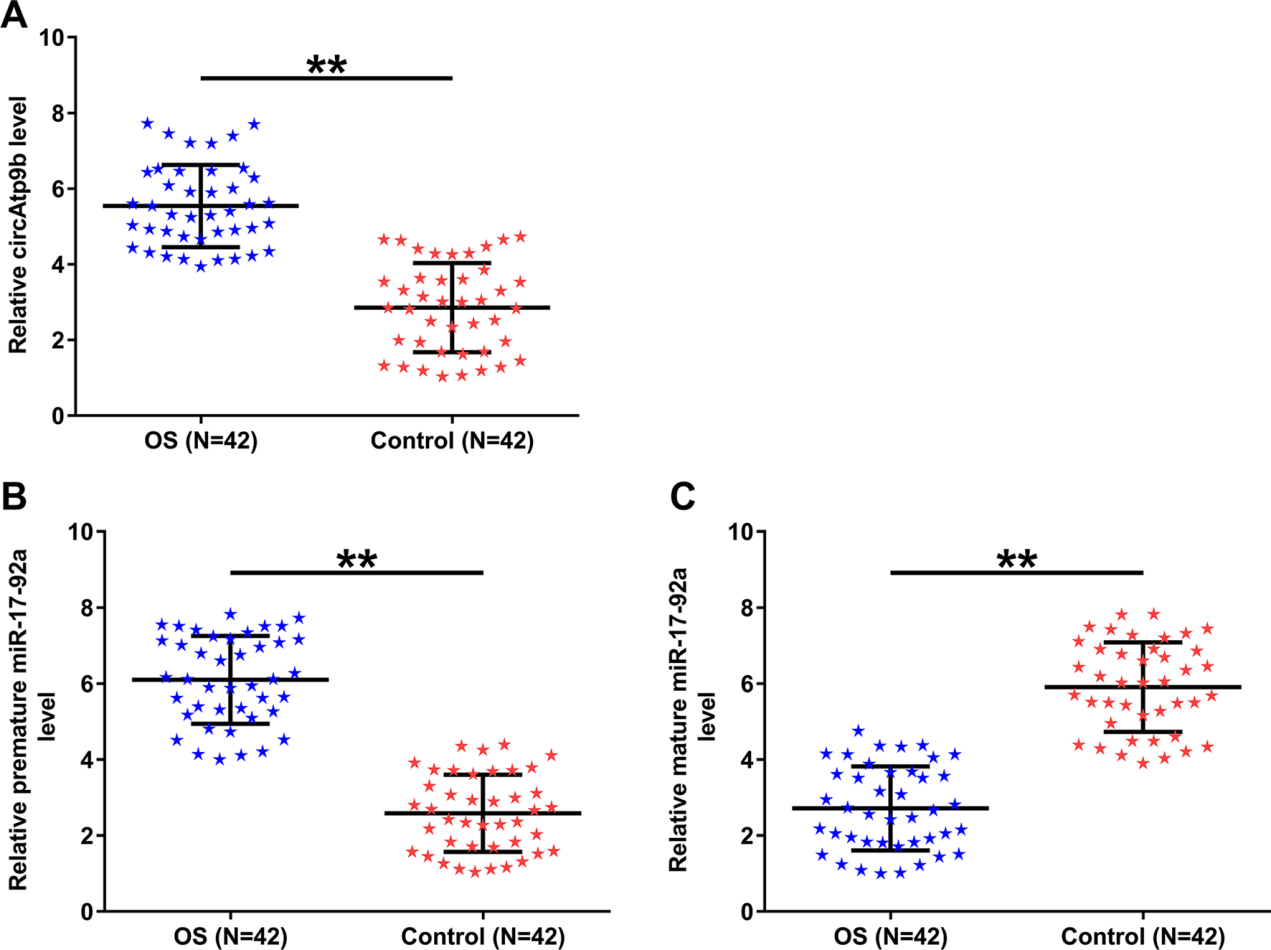
Analysis of circAtp9b and miR-17-92a expression in OS. Analysis of circAtp9b (**A**), premature miR-17-92a (**B**), and mature miR-17-92a (**C**) in samples of plasma from 42 OS patients and 42 controls was performed with RT-qPCRs. **, *p* < 0.01

The expression of circAtp9b (Fig. 1A), premature miR-17-92a (Fig. 1B), and mature miR-17-92a (Fig. 1C) in plasma samples of the 42 OS patients and 42 controls was detected by RT-qPCRs. OS patients exhibited upregulated expression of circAtp9b and premature miR-17-92a, and downregulated expression of mature miR-17-92a (*p* < 0.01). Therefore, upregulation of circAtp9b and the inhibited maturation of miR-17-92a are likely involved in OS.

### The role of circAtp9b in regulating the expression of mature and premature miR-17-92a

**Fig. 2:**
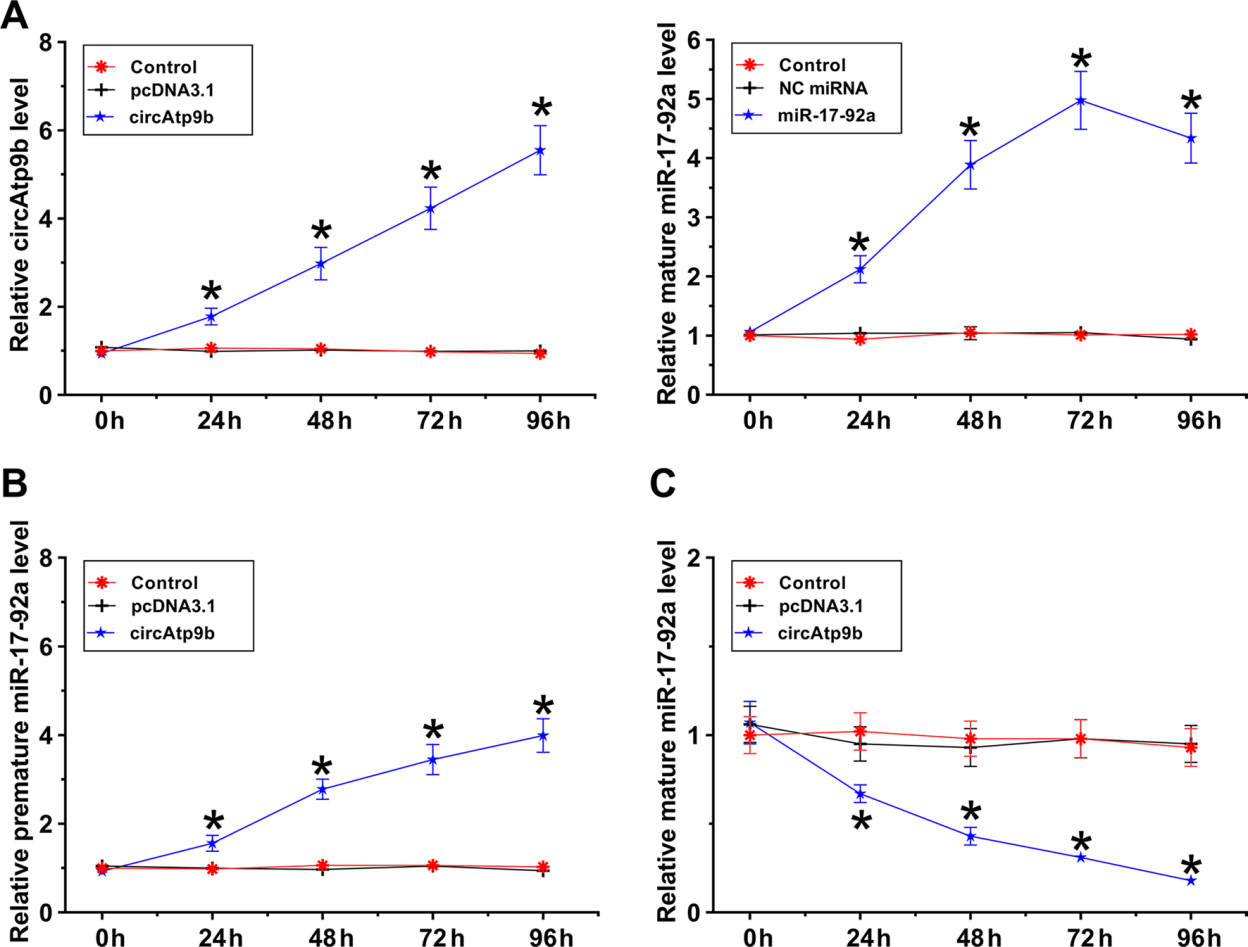
Analysis of the role of circAtp9b in the expression of mature and premature miR-17-92a. Osteoblasts were overexpressed with circAtp9b or miR-17-92a, followed by the confirmation of transfections every 24 h until 96 h (**A**). The role of circAtp9b in regulating the expression of premature miR-17-92a (**B**) and mature miR-17-92a (**C**) was explored by RT-qPCR. *,* p* < 0.01

Osteoblasts were overexpressed with circAtp9b or miR-17-92a, followed by the confirmation of overexpression every 24 h until 96 h (Fig. 2A , *p* <  0.05). It was observed that overexpression of circAtp9b increased the expression levels of premature miR-17-92a (Fig. 2B , *p* < 0.05), but decreased the expression levels of mature miR-17-92a (Fig. 2C , *p* < 0.05). Therefore, circAtp9b could suppress the maturation of miR-17-92a in osteoblasts.

### The subcellular location of circAtp9b in osteoblasts and the direction interaction of circAtp9b with premature miR-17-92a

**Fig. 3:**
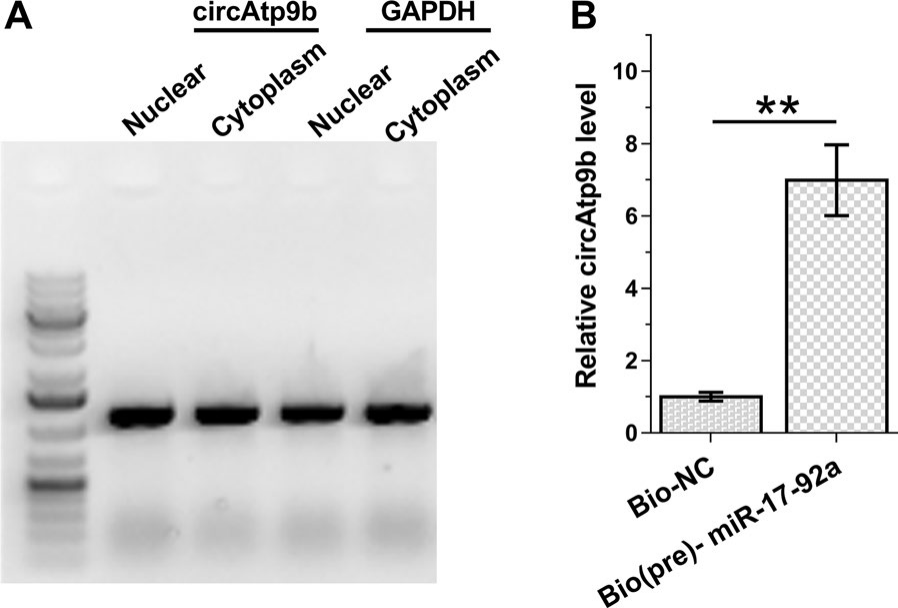
Analysis of the subcellular location of circAtp9b in osteoblasts and the direction interaction of circAtp9b with premature miR-17-92a. Subcellular fractionation assay was performed to explore the subcellular location of circAtp9b in osteoblasts (**A**). RNA pull-down assay was performed to explore the direct interaction of circAtp9b with premature miR-17-92a (**B**). **, *p* < 0.01

Subcellular fractionation assay was performed to explore the subcellular location of circAtp9b in osteoblasts. It was observed that circAtp9b could be detected in both nuclear and cytoplasm samples of osteoblasts (Fig. 3A). RNA pull-down assay was performed to explore the direct interaction between circAtp9b and premature miR-17-92a. Compared to Bio-NC pull-down sample, Bio(pre)- miR-17-92a pull-down sample exhibited significantly higher expression levels of circAtp9b (Fig. 3B , *p* < 0.01). Therefore, circAtp9b in nuclear may interact with premature miR-17-92a.

### The role of circAtp9b and miR-17-92a in the apoptosis of osteoblasts

**Fig. 4:**
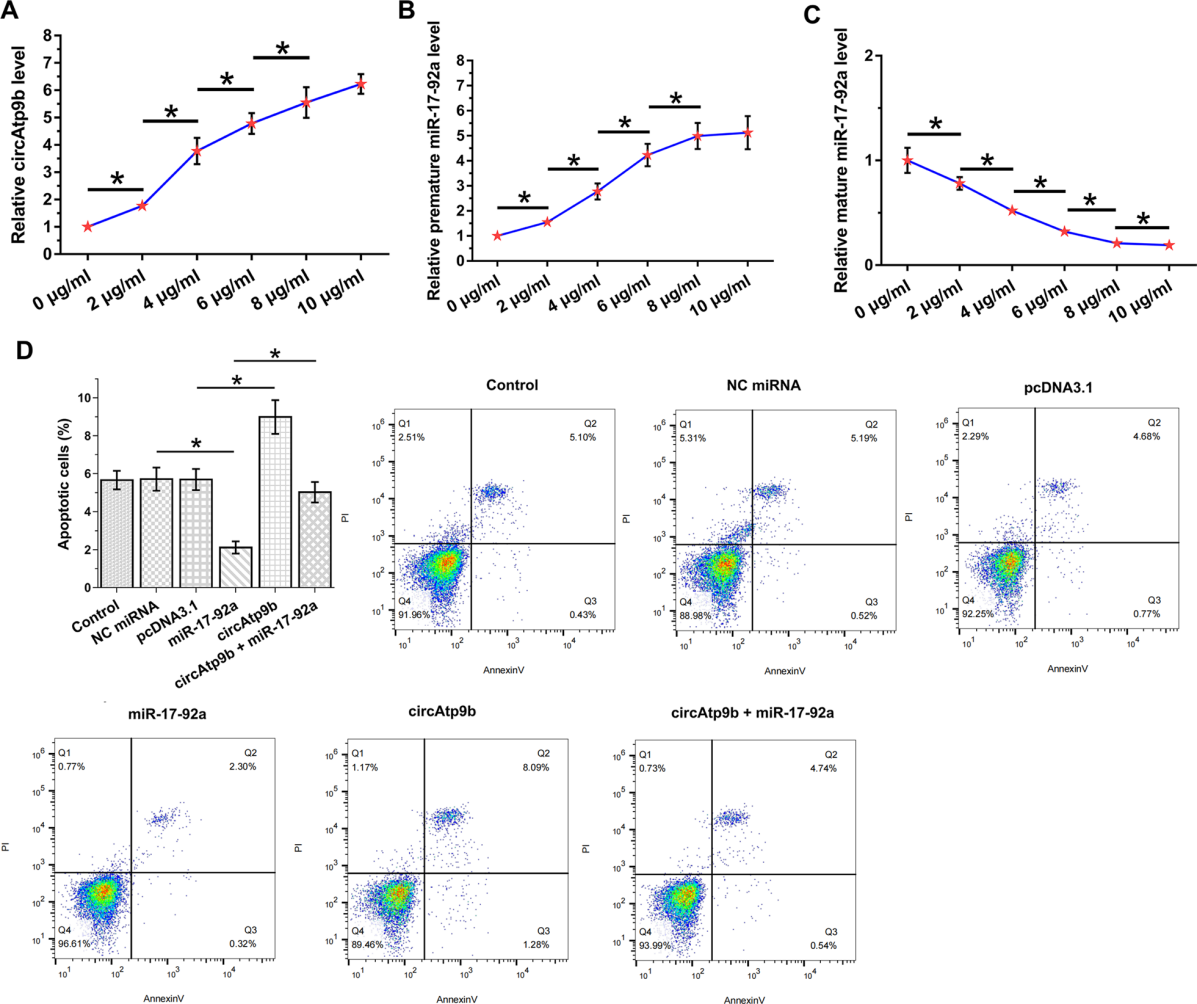
Analysis of the role of circAtp9b and miR-17-92a in the apoptosis of osteoblasts. Osteoblasts were treated with medium containing 0, 2, 4, 6, 8 and 10 μg/ml LPS was performed for 48 h to achieve LPS treatment, followed by the determination of the expression of circAtp9b (**A**), premature miR-17-92a (**B**) and mature miR-17-92a (**C**). Cell apoptosis assay was performed to explore the role of circAtp9b and miR-17-92a in the apoptosis of osteoblasts (**D**). CircAtp9b + miR-17-92a, cells co-overexpressed with circAtp9b and mature miR-17-92a. *,* p* < 0.05

LPS-treated osteoblast is a widely used cell model of OS. To this end, osteoblasts were treated with medium containing 0, 2, 4, 6, 8 and 10 μg/ml LPS for 48 h to achieve LPS treatment. LPS upregulated circAtp9b (Fig. 4A, *p* < 0.05) and premature miR-17-92a (Fig. 4B, *p* < 0.05), and decreased the expression levels of mature miR-17-92a (Fig. 4C, *p* < 0.05) in osteoblasts. From 0 to 10 μg/ml, LPS altered the expression of circAtp9b, premature miR-17-92a and mature miR-17-92a in a dose-dependent manner, and the effects of LPS-induced gene expression reached the peak values at 8 or 10 μg/ml (based on the curves). Cell apoptosis assay was then performed with 10 μg/ml LPS treatment to explore the role of circAtp9b and miR-17-92a in the apoptosis of osteoblasts. CircAtp9b increased cell apoptosis, and miR-17-92a decreased cell apoptosis. Moreover, circAtp9b reduced the effects of miR-17-92a on the apoptosis of osteoblasts induced by LPS (Fig. 4D, *p* < 0.05). In addition, circAtp9b siRNA silencing was also performed (Additional file 1: Fig. S1A, *p* < 0.01). Cell apoptosis after 10 μg/ml LPS treatment was also analyzed. It was observed that circAtp9b significantly decreased cell apoptosis (Additional file 1: Fig. S1B, *p* < 0.01).

## Discussion

This study explored the role of circAtp9b and miR-17-92a in OS and the interactions between them. Our results showed that circAtp9b was upregulated in OS and it may suppress the maturation of miR-17-92a in osteoblasts to increase the apoptosis of osteoblasts.

A recent study revealed the enhancing effects of circAtp9b on inflammatory responses, and knockdown of circAtp9b alleviates inflammation induced by LPS [15]. Moreover, it was reported that circAtp9b regulates the progression of OS by targeting miR-138-5p [18]. However, the role of circAtp9b in OS is unclear. This study is the first to report the upregulation of circAtp9b in OS. In addition, LPS treatment increased the expression levels of circAtp9b in osteoblasts in a dose-dependent manner. Therefore, the upregulation of circAtp9b in OS is likely induced by LPS. Osteoblasts are cells from bone marrow and mainly responsible for the formation of new bones, which are made of collagen and many other proteins [19]. In effect, increased apoptosis of osteoblasts is involved in the progression of OS. In this study, we showed that overexpression of circAtp9b increased the apoptosis of osteoblasts induced by LPS. Therefore, circAtp9b may participate in OS by increasing the apoptosis of osteoblasts induced by LPS.

A recent study characterized miR-17-92a as an estrogen-inducible miRNA [17]. In addition, overexpression of miR-17-92a targets Bim to inhibit the apoptosis of osteoblast [17]. This study showed the upregulation of miR-17-92a in OS and confirmed the inhibitory effects of this miRNA on LPS-induced apoptosis of osteoblasts. However, the upstream regulator of miR-17-92a in the apoptosis of osteoblasts induced by LPS is unclear. In this study, we showed that circAtp9b could directly interact with premature miR-17-92a. Interestingly, overexpression of circAtp9b increased the expression levels of premature miR-17-92a, but significantly decreased the expression levels of mature of miR-17-92a. Moreover, circAtp9b was detected in both nuclear and cytoplasm samples of osteoblasts. We therefore speculated that circAtp9b could sponge premature miR-17-92a and suppress its maturation.

The present study showed that circAtp9b may suppress the maturation of miR-17-92a to promote the apoptosis of osteoblasts in OS. However, this conclusion remains to be verified by in vivo animal model experiments, which are hard to be carried out. Our findings suggested that the circAtp9b/miR-17-92a axis may serve as a potential target to treat OS. With the increased understanding of the potentials of non-coding RNAs in the treatment of human diseases [20–22], novel anti-OS approaches are expected to be developed in near future. However, clinical trials are needed to test our hypothesis.

## Conclusion

CircAtp9b was upregulated in OS, and it may suppress the maturation of miR-17-92a in osteoblasts to increase the apoptosis of osteoblasts, thereby accelerating the progression of OS.

## Supplementary Information

**Additional file 1: Figure S1**. The role of circAtp9b siRNA silencing in the apoptosis of osteoblasts. CircAtp9b siRNA silencing was also achieved in osteoblasts (A). Cell apoptosis after 10 μg/ml LPS treatment was also analyzed (B). *, *p* < 0.05.

## Data Availability

The analyzed data sets generated during the study are available from the corresponding author on reasonable request.

## Abbreviation

OS: Osteoporosis
