# Supplementary figures and images for: LPS-inducible circAtp9b is highly expressed in osteoporosis and promotes the apoptosis of osteoblasts by reducing the formation of mature miR-17-92a
Source: J Orthop Surg Res. 2022 Mar 28;17:193. doi: 10.1186/s13018-022-03072-x (PMC8962610; doi:10.1186/s13018-022-03072-x)

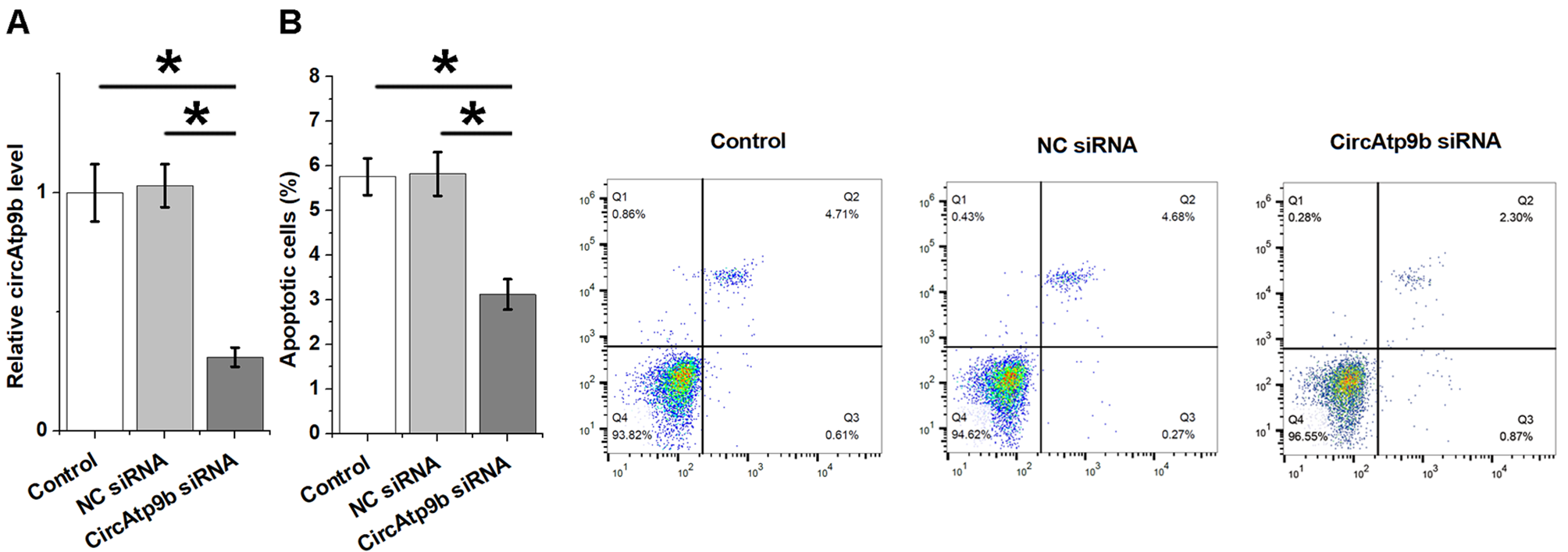

Supplement: Supplementary file 1 — Additional file 1: Figure S1. The role of circAtp9b siRNA silencing in the apoptosis of osteoblasts. CircAtp9b siRNA silencing was also achieved in osteoblasts (A). Cell apoptosis after 10 μg/ml LPS treatment was also analyzed (B). *, p < 0.05. [file 13018_2022_3072_MOESM1_ESM.tif]
